# Cross-Cultural Adaptation and Validation of the Brazilian Portuguese Version of an Observational Measure for Parent–Child Responsive Caregiving

**DOI:** 10.3390/ijerph18031246

**Published:** 2021-01-30

**Authors:** Alessandra Schneider, Michelle Rodrigues, Olesya Falenchuk, Tiago N. Munhoz, Aluisio J. D. Barros, Joseph Murray, Marlos R. Domingues, Jennifer M. Jenkins

**Affiliations:** 1 Department of Applied Psychology and Human Development, University of Toronto, Toronto, ON M5S, Canada; alessandra.schneider@mail.utoronto.ca (A.S.); michelle.rodrigues@mail.utoronto.ca (M.R.); 2 Ontario Institute for Studies in Education, University of Toronto, Toronto, ON M5S, Canada; olesya.falenchuk@utoronto.ca; 3 Faculty of Psychology, Federal University of Pelotas, Pelotas 96010900, Brazil; tyagomunhoz@hotmail.com; 4 Postgraduate Program in Epidemiology, Federal University of Pelotas, Pelotas 96010900, Brazil; abarros.epi@gmail.com (A.J.D.B.); j.murray@doveresearch.org (J.M.); 5 Human Development and Violence Research Centre, Federal University of Pelotas, Pelotas 96010900, Brazil; 6 Postgraduate Program in Physical Education, Federal University of Pelotas, Pelotas 96010900, Brazil; marlosufpel@gmail.com

**Keywords:** responsive caregiving, parent–child interaction, observational measurement, thin slice methodology, low- and middle-income countries, Brazil

## Abstract

Responsive caregiving is the dimension of parenting most consistently related to later child functioning in both developing and developed countries. There is a growing need for efficient, psychometrically sound and culturally appropriate measurement of this construct. This study describes the cross-cultural validation in Brazil of the Responsive Interactions for Learning (RIFL-P) measure, requiring only eight minutes for assessment and coding. The cross-cultural adaptation used a recognized seven-step procedure. The adapted version was applied to a stratified sample of 153 Brazilian mother–child (18 months) dyads. Videos of mother–child interaction were coded using the RIFL-P and a longer gold standard parenting assessment. Mothers completed a survey on child stimulation (18 months) and child outcomes were measured at 24 months. Internal consistency (α = 0.94), inter-rater reliability (*r* = 0.83), and intra-rater reliability (*r* = 0.94) were all satisfactory to high. RIFL-P scores were significantly correlated with another measurement of parenting (*r*’s ranged from 0.32 to 0.47, *p* < 0.001), stimulation markers (*r* = 0.34, *p* < 0.01), and children’s cognition (*r* = 0.29, *p* < 0.001), language (*r* = 0.28, *p* < 0.001), and positive behavior (*r* = 0.17, *p* < 0.05). The Brazilian Portuguese version is a valid and reliable instrument for a brief assessment of responsive caregiving.

## 1. Introduction

Responsive caregiving is a key element in fostering young children’s developmental potential [1,2,3,4]. This special type of caregiving integrates sensitivity (defined as the caregiver´s ability to notice, interpret, and respond appropriately to an infant´s signals, needs, and internal state [5]) and stimulation (described as expanding and building on a child’s interest by talking, pointing and demonstrating in a developmentally appropriate way that supports early learning [6]). These attuned and reciprocal interactions—previously operationalized as cognitive sensitivity [7] or responsive stimulation [3]—have been found to predict cognitive [4,8,9], socioemotional [10,11], and brain development in young children [12]. This aspect of parenting is best assessed observationally as caregivers can only report on responses to signals that they notice and not those they miss or misinterpret [13,14].

The Responsive Interactions for Learning (RIFL) measure combines the well-understood concepts of sensitivity and stimulation in a brief, observational tool that can be used to assess responsive caregiving at the population level. It has been shown to have good reliability and validity in mothers, fathers, and siblings and is referred to as RIFL-P for parents [15] and RIFL-S for siblings [7,16]. The coding scheme for this instrument uses a thin-slice methodology proposed by Ambady [17], who argued that when a construct is well articulated, it can be accurately, intuitively, and rapidly rated. Thin-slice ratings have been shown to have similar psychometric properties to labor-intensive coding schemes [7,15].

Considering that most instruments measuring parental responsivity have been developed in Western countries based on middle-class samples [18], one cannot presume that specific behaviors observed for those families and assessed by those instruments are generalizable across cultures. To overcome this, the field recommends adaptation of instruments with documented validity rather than the development of new ones since cross-cultural adaptation is faster, easier, and less expensive [19,20]. Experimental and correlational studies performed in Brazil have shown cultural evidence of the importance of the construct of responsivity/sensitivity in the Brazilian society [21,22,23].

Given the public health importance of early responsivity to child development, and considering that responsive caregiving is the cornerstone of successful early childhood development (ECD) interventions [24,25], there is an urgent need for valid and reliable measures appropriate for use in large-scale studies. Screening of parental responsivity at the level of population groups could aid in identifying those caregivers who may benefit from parenting programs. There is also growing recognition of the need to integrate behavioral services into primary care [26] to strengthen early identification and access to appropriate interventions [27]. This is especially relevant in Brazil, as this country has been implementing massive ECD home visiting programs among disadvantaged families [28,29] and parent training programs to coach caregivers on positive parent–child interactions [30].

The aim of the study was to describe the cross-cultural adaptation process and validation of the Brazilian Portuguese RIFL-P, thus providing a culturally adapted, validated, and appropriate observational instrument to assess responsive caregiving in Brazilian parent–child dyads.

## 2. Materials and Methods

The University of Toronto Research Ethics Board, as well as the Ethics Committee of the Medical School of the Federal University of Pelotas approved the study. All participants signed an informed consent form before being enrolled in this study.

### 2.1. Participants

Participants of phase 1 (cross-cultural adaptation) and phase 2 (testing the new measure) were different. For phase 1, nineteen participants took part, including supervisors and home visitors from the *Primeira Infância Melhor* program (*N* = 17) and two child health university professors.

The phase 2 study was based on a subsample of the 2015 Pelotas Birth Cohort Study [31] when the children were 18 months old. The target sample size was *N* = 155, based on budgetary constraints and practice in the field [32,33]. The Pelotas cohort used demographic data collected within two days of the child’s birth to identify a subpopulation of children eligible for the current parenting study. Three hundred and ninety-five families satisfied the selection criteria (full-term, singleton, normal birth weight, and aged between 17 and 18 months during a six-week data collection period). Families were stratified by wealth quintiles according to household assets assessed at the child’s birth and recruited until the target sample size was achieved. Recruiting stopped after 178 families were contacted, 23 families refused (13%) and the target sample of *N* = 155 was achieved. Observational data were collected for 155 mother–child dyads. Two film clips were excluded for technical reasons (duration of less than 5 min and third-party interference in the task). The final sample of 153 dyads was determined to be optimal based on the proposed analyses and expected results [34]. Mean child age was 17.9 months (SD = 0.27), mean gestational age was 39.4 weeks (SD = 1.23; range = 37–41.9 weeks). Females outnumbered males (females = 55.5%, males = 44.5%). The participating families’ socioeconomic levels were represented equally in the upper four quintiles with about 22% each, while the first quintile (the poorest) accounted for about 12% of the children. Children in the 2015 Pelotas Birth Cohort were followed up at 24 months of age, and developmental outcomes were collected [35]. The relationship between our new parental responsivity measure and children’s developmental outcomes could therefore be examined in the 153 families of our sample.

### 2.2. The Responsive Interactions for Learning Measure, Version for Parents

The RIFL-P (previously known as Cognitive Sensitivity) is a unidimensional 11-item observational instrument designed to provide a rapid assessment of the extent to which a parent identifies and responds, incorporating sensitivity and stimulation, to the feelings and thoughts of the child with whom they are interacting. The RIFL-P measures three interconnected skills of the caregiver—(i) communicative clarity (providing meaningful verbal/nonverbal inputs to the child and fostering of shared understanding of the goals of the task); (ii) mind-reading (thinking about what the child knows and understands); and (iii) mutuality building (promoting reciprocity)—through a challenging task that elicits cooperation. Assessment uses a thin slice methodology and takes around eight minutes to administer and code (five minutes of observation of interaction, three minutes to code). After watching a 5-min video recording just once, raters apply codes to each of the 11 items using a five-point Likert scale, ranging from 1 (“Not at all true”) to 5 (“Very true”). A mean of the 11 items is calculated, yielding a composite score of responsivity that can range from 1 to 5. Currently, the training of RIFL-P raters is completed in less than eight hours through a password-protected, open-source online asynchronous course offered by the University of Toronto, which is available in English, Portuguese, and Spanish. Psychometric properties of the original instrument were found to be strong (inter-rater reliability (IRR) was α = 0.84; internal consistency of the scale was α = 0.92) [15]. RIFL scores assessed both for parents and siblings have been found to be associated with contextual risk (inversely), traditional measures of maternal sensitivity, and a range of child outcomes, including receptive vocabulary, executive functioning, theory of mind, and academic achievement [7,15,36].

### 2.3. Phase 1: Cross-Cultural Adaptation of the RIFL-P

**Figure 1:**
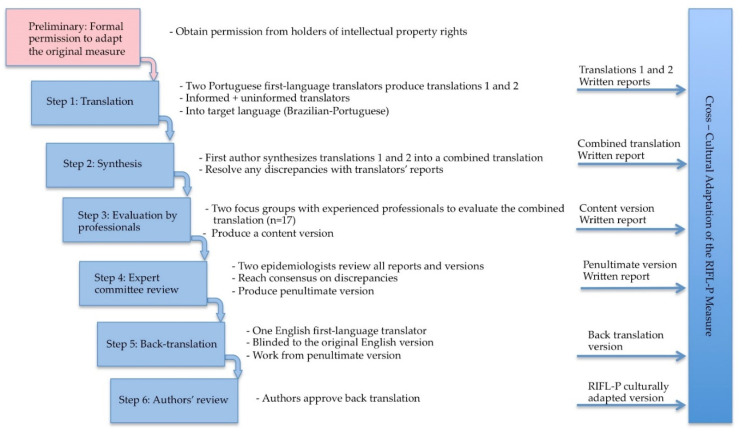
Step sequence for the cross-cultural adaptation process—the Brazilian Portuguese version of the RIFL-P measure (adapted from Beaton et al. [19]).

Phase 1 was the cross-cultural adaptation of the RIFL-P from the source to the target language. Prior to conducting this phase, written permission from the developers of the original measure was obtained. A well-established method [19,37] was used for this phase, based on six steps (see Figure 1) that maximize the level of semantic, idiomatic, conceptual, and experiential equivalence achieved between the original and adapted versions of the instrument.

At the end of each step, a written report and/or an updated version of the instrument were produced and used to guide the next step. The focus groups and the formation of an expert committee ensured conceptual and functional content validity [38]. The first author (AS) coordinated the two 2-h group sessions with nine and eight participants each. Participants were asked to read and discuss all the items in the scale, the response options, and the extent to which the wording was clear and comprehensible. They collectively rated the comprehensibility of the scale on a three-point scale. They were asked to consider whether the items were representative of the parenting of Brazilian parents and to raise any other important aspects of parenting that came to mind. Back translation was elaborated with a double purpose, i.e., highlighting translation deficiencies and allowing for review of possible cultural differences by the original developers. Back translation is viewed as an additional quality control check [19,39]. It is also essential in a cross-cultural adaptation of an observational measure to contextualize the coding in the new culture [32], as described below.

### 2.4. Phase 2: Testing the New Measure

This phase included the collection and analysis of 155 videos of mother–child interaction. This phase had two components: Coder Training and Full Psychometrics. The purpose of Coder Training was to train the second coder in the reliability criteria and to elaborate the coder manual (for future training of coders) with specific information about the Brazilian context and parenting. This was done with the first 39 dyads from the full sample. The purpose of the Full Psychometrics component was to establish the reliability and validity of the Brazilian Portuguese RIFL-P measure (namely, *Interações Responsivas para a Aprendizagem*).

#### 2.4.1. Measures

##### The RIFL-P (Brazilian Portuguese Version)

Mothers were given a task to do with their children. The mother sat on a yoga mat and played with her child for five minutes. She was shown pictures of patterns on the shape and color sorter of varying degrees of difficulty and asked to encourage the child to make the patterns with her. The goal was to elicit maternal behaviors and speech related to helping and teaching when the task was slightly too developmentally challenging for the child. Following training on the scale, and after watching the recording once, raters applied codes to each of the 11 items using a five-point Likert scale, ranging from 1 (“Not at all true”) to 5 (“Very true”). The task plus coding took around eight minutes.

##### Parenting Interactions with Children: Checklist of Observations Linked to Outcomes, PICCOLO (Brazilian Portuguese Version)

PICCOLO is a strengths-based checklist of 29 observable behaviors used to assess positive parenting interactions with children aged 10 to 47 months [40]. PICCOLO items are clustered in four domains with seven to eight items per domain: (a) affection (warmth, physical closeness, and positive expressions toward the child); (b) responsiveness (responding sensitively to a child’s cues, needs, interests, and behaviors); (c) encouragement (active support of play, exploration, curiosity, skills, and creativity); and (d) teaching (shared conversations and play, cognitive stimulation, explanations, and questions). After watching a 10-min film clip once, raters coded items on a three-point ordinal scale from “Absent”, or not seen, to “Clearly” seen. The task plus coding took around 45 min. Psychometric properties of the original instrument have been found to be strong (IRR ranged between *r* = 0.74 for the responsiveness domain and *r* = 0.80 for the affection domain; Cronbach’s α coefficient = 0.91 for the total PICCOLO score (ranging from α’s of 0.75 for the responsiveness domain to 0.80 for the teaching domain)) [40], as have those of the PICCOLO Brazilian Portuguese version (IRR ranged between *r* = 0.63 for the encouragement domain and *r* = 0.77 for the affection domain; Cronbach’s α coefficient = 0.94 for the total PICCOLO score (ranging from α’s of 0.79 for the affection and teaching domains to 0.86 for the responsiveness and encouragement domains)) [41].

##### INTERGROWTH-21st Neurodevelopment Assessment

The INTERGROWTH-21st Neurodevelopment Assessment (INTER-NDA) is a valid and reliable international standardized screening assessment of early child development at 2 years of age [42]. This multidimensional measure provides a comprehensive, rapid assessment of cognition, fine and gross motor skills, language, and positive and negative behavior for children aged 22–30 months. Its 37 items are administered in approximately 15 min using a combination of psychometric techniques, such as direct administration, concurrent observation, and caregiver reports. For all INTER-NDA domains, except for negative behavior, higher scores reflect better outcomes. The INTER-NDA has demonstrated good to acceptable agreement with the Bayley Scales of Infant and Toddler Development, Third Edition [43]. The INTER-NDA is designed to be applied by nonspecialists to high-, middle-, and low-income populations. The INTER-NDA instrument was used to evaluate the development of 3776 children aged 24 months from the 2015 Pelotas Birth Cohort Study in Southern Brazil [35].

##### Five Stimulation Markers

This scale was developed by Barros and colleagues [44] to examine the extent to which children experienced a cognitively stimulating environment. Mothers answered no/yes (no = 0, yes = 1) to the following questions about their children’s activities in the past week: whether someone read or told a story to the child; whether the child went to a park or playground; whether the child had a story book; whether the child watched TV; whether the child visited anyone’s house. The responses for the five items were summed, resulting in a scale from 0 to 5 points. The scale has been found to be related to child development outcomes in Brazil, particularly amongst children whose mothers are low in education [44].

#### 2.4.2. Procedures

Data collection for phase 2 included a home visit to film mother–child interactions (10 min PICCOLO task followed by the 5 min RIFL-P task) when children were around 18 months old. The mothers answered a one-page questionnaire about the five stimulation markers before the home visit was terminated. The filmed interactions (collected at 18 months) were coded and analyzed in relation to demographic data (collected up to two days after delivery, in the perinatal period) of the 2015 Pelotas Birth Cohort Study and developmental data collected when children reached 24 months of age.

#### 2.4.3. Data Coding Procedures

The first (AS) and fourth (TNM) authors, trained by expert coders, scored the RIFL-P videos. Raters were trained until the inter-rater reliability of *r* > 0.80 was reached and then coded the remaining videos independently. Reliability testing was conducted using every fourth film throughout data coding. Following the submission of reliability scores, discrepancies were discussed to minimize rater drift. Scale developers were consulted on three occasions.

### 2.5. Data Analysis

Investigation of psychometric properties of the adapted RIFL-P instrument included inter- and intra-rater reliability (Pearson correlation coefficient), item-level descriptive statistics, and confirmatory factor analysis (CFA). The internal consistency of the adapted version was computed using Cronbach’s alpha.

To examine convergent validity, the composite score of RIFL-P was correlated with the scores of the PICCOLO Brazilian Portuguese version, Five Stimulation Markers, and INTER-NDA scales. Discriminant validity was tested by examining the relationship between gender and the RIFL-P scores and no significant association was expected. Different types of correlation were used dependent on variable type (Pearson product-moment correlation for continuous variables, point-biserial correlation for binary and continuous variables, and Spearman’s correlation for ordinal variables). Statistical analyses were conducted using the Statistical Package for Social Sciences (SPSS, Inc., Chicago, IL, USA) for Windows (version 21.0; Microsoft Corporation, Redmond, WA, USA), and the Stata^®^ version 13.0 software.

## 3. Results

### 3.1. Phase 1: Cross-Cultural Adaptation of the RIFL-P

Three key adaptations were made during development of the Brazilian Portuguese RIFL-P version. First, the term “parent” was replaced by “caregiver”. Second, in Portuguese, “mind reading” was translated as “thought reading” (*leitura do pensamento*) in order to preserve conceptual equivalence. Third, definitions of the scoring rubrics of the adapted RIFL-P version were expanded to include a specific rubric for score 3 (“Sometimes true/I partially agree”) and descriptions of scores 1 and 5 of the Likert scale gained one more expression (i.e., 1 = “Not at all true/I totally disagree”; 5 = “Very true/I totally agree”). The adapted RIFL-P coding sheet and manual, in Brazilian Portuguese, are presented as Appendix A and Appendix B respectively.

### 3.2. Phase 2: Testing the New Measure

#### 3.2.1. Coder Training

Additional guidelines for Brazilian coders (i.e., examples of culture-specific behaviors) were elaborated for five of the 11 items to adapt the measure, both culturally and psychometrically, for use with the local population. Three items were coded infrequently (1, 3, and 8), meaning that what was average for those behaviors for Canadian mothers was not average for Brazilian mothers. In order to ensure the underlying normal distribution on all items in the adapted RIFL-P measure, the scoring rubrics for those three items were softened in the Portuguese Brazilian version.

#### 3.2.2. Full Psychometrics

Descriptive statistics for the RIFL-P items in the Brazilian sample are presented in Table 1. The table shows that the least frequently observed item in the Brazilian sample was item 8, namely “This parent is good at rephrasing what the child does not understand” and the most frequently observed behavior was item 1 “This parent gives clear and specific verbal directions”.

##### Reliability

Inter-rater reliability of the total scale score was high (*r* = 0.83), well above the acceptable minimum of 0.70 [45]. Intra-rater reliability, or rating consistency by the same rater two weeks apart, was also high (*r* = 0.94).

##### Factorial Structure

Confirmatory factor analysis was conducted to investigate whether the unidimensional structure of the original RIFL-P instrument applied to the Portuguese version. Model fit for the one-dimensional CFA model was less than optimal (RMSEA = 0.12; CFI = 0.93; TLI = 0.92; cut-off recommendations for acceptable fit require RMSEA < 0.06; CFI > 0.95; TLI > 0.95) [46]. A likelihood ratio test comparing the CFA model to the saturated model was significant, χ^2^(44) = 143.55, *p* < 0.001. Investigation of the modification indices showed very high correlations across three pairs of items (3 and 8, 1 and 6, and 8 and 10; modification indices were 31.95, 16.88, and 13.52, respectively). Consequently, the insufficient model fit in the CFA analysis was not due to deficient relationships among the items but rather to potential redundancies among some of them. Once the CFA model was modified to take these correlations into account, the model fit indices were high (RMSEA = 0.05; CFI = 0.97; TLI = 0.96). All 11 items had high standardized factor loadings on a single factor (ranging from 0.61 to 0.90, shown in Table 2). The percentage of variance explained by the latent factor in each item (R2) ranged between 0.37 and 0.81. This unidimensional structure was further confirmed with the internal consistency analysis. The Cronbach’s alpha was 0.94, with item-total correlations ranging between 0.61 and 0.88.

Based on the results of psychometric analyses, composite RIFL-P scores were computed as the mean of the 11 items. The mean Brazilian Portuguese RIFL-P score was 2.62 (SD = 0.81), ranging between 1.09 and 4.82. Comparable scores for the original measure, in a sample of Canadian mother–child dyads, had a mean of 3.24 (SD = 0.70), with a minimum score of 1.18 and a maximum score of 4.91 [15]. The differences between the average RIFL-P scores in the Brazilian Portuguese and Canadian samples was statistically significant, t(437) = 8.36, *p* < 0.001, with a large effect size (Cohen’s d = 0.82). RIFL-P composite scores were positively correlated with family socioeconomic status (Spearman’s *ρ* = 0.46, *p* < 0.001).

##### Convergent and Discriminant Validity

The convergent validity analyses showed that the adapted RIFL-P was significantly associated with all four Brazilian Portuguese PICCOLO domains (*r* = 0.32 for affection; *r* = 0.37 for responsiveness; *r* = 0.41 for encouragement; and *r* = 0.47 for teaching, *p* < 0.001) and the PICCOLO total score (*r* = 0.44, *p* < 0.001). In addition, the adapted RIFL-P was significantly associated with the Five Stimulation Markers (*r* = 0.34, *p* < 0.01) and a range of child outcomes assessed by the INTER-NDA, including cognition (*r* = 0.29, *p* < 0.001), language (*r* = 0.28, *p* < 0.001), and positive behavior (*r* = 0.17, *p* < 0.05). Its association with fine motor skills (*r* = 0.16, *p* = 0.055) was borderline significant and it was not significantly associated with gross motor skills (*r* = 0.13, *p* = 0.110) or negative behavior scores (*r* = −0.001, *p* = 0.992). With respect to discriminant validity, RIFL-P was not expected to correlate with gender (*r* = −0.22, *p* < 0.01) but it did. The RIFL-P scores for boys were lower than for girls.

## 4. Discussion

Responsive caregiving is a dimension of parenting in the early childhood years that is consistently related to subsequent cognitive and socioemotional aspects of functioning [2,47,48]. This aspect of caregiving is modifiable. Results from randomized controlled trials in developed and developing countries show that it is possible to improve this aspect of parenting, that doing so improves children’s developmental outcomes, and that it may be the modifiable risk factor with the strongest effect on later brain development [3,49,50]. Although marked improvements to long-term child outcomes have now been demonstrated for parental responsivity interventions [4], moving from efficacy to effectiveness has proven to be a substantial challenge. If scalability is to be achieved, it is essential to have an instrument that can measure parental responsivity. Unfortunately, however, observational measures are the only ones that show good validity and most previous measures take a long time to administer and code. On the other hand, the literature suggests that adaptation of instruments that have shown reliability and validity evidence is more advantageous in LMIC than novel instrument development [19].

The cross-cultural adaptation of RIFL-P involved gold standard methodology to avoid cultural bias and the measure was successfully adapted to Portuguese and the Brazilian context. The adapted RIFL-P measure showed strong psychometric properties, mirroring the findings from the original instrument. As it is efficient, only taking eight minutes to assess and code, it is a measure that can be used at the population level. Since the sample was based on a stratified sample from a population cohort in one city of Brazil and refusal rates from the cohort were low, a high level of representativeness was achieved. A recent global effort led by the World Health Organization and UNICEF to monitor the Nurturing Care Framework’s responsive caregiving component only provided proxy indicators (e.g., parental mental health, childcare availability, parental support) [51]. To make real progress in this realm, it is imperative to assess responsive interactions at the population level with measures, such as RIFL-P, that are psychometrically strong and quick to train, administer, and code. An online course in three languages is available to researchers or parenting professionals free of charge by contacting the first or last authors.

Four findings are worthy of further comment. First, the mean score for responsive interactions in Brazil was lower than that found in Canada. This was expected given the results of earlier studies in which the parenting of Latino mothers has been found to be more directive and controlling [52], with a lack of emphasis on understanding the child’s internal goals and need for autonomy [53]. Of course, cultures differ in their values, customs, and beliefs [54], with the result that some parental behaviors receive less attention than others [55]. Regarding childrearing practices, Latino mothers tend to value obedience and politeness, physically guide their toddler´s actions, attribute less importance to children´s autonomy, and report greater use of discipline as a teaching method when compared to European American mothers of the same socioeconomic status [56]. Given that parental responsivity (although lower in Brazil) was found to relate to children’s developmental outcomes, this does suggest that improving parental responsivity should be an important policy goal in Brazil. Second, in this study, Brazilian mothers were more responsive to girls than to boys, according to the RIFL-P scores. This finding should be cautiously interpreted as the same gender difference was not observed based on PICCOLO scores and a meta-analysis of parenting as a function of gender reported no significant differences [57]. An examination of this in future studies in Brazil is encouraged. Third, for purposes of criterion validity, we examined relationships with child outcomes, as well as other indices of parenting and contextual risk. Findings showed that both parenting measures were significantly related (i.e., RIFL-P and PICCOLO scores), as well as RIFL-P scores and a measure of parenting stimulation [44], and the strongest relationships with child outcomes were observed for the cognitive and language domains, which is in line with previous findings for the RIFL-P in a Canadian sample [15]. RIFL-P scores, both in Brazil and Canada, have been found to be associated with contextual risk (inversely). Fourth, as in previous work, low socioeconomic status (SES) was found in the current study to be associated with lower levels of parental responsivity. The association between socioeconomics and parenting has been widely reported [58] with evidence that economic hardship leads to parental emotional distress which impairs parenting [59]. It is also notable that economic hardship does not operate on its own. SES clusters risk [60] such that children are exposed to multiple challenges that compromise development (e.g., maternal depression, unemployment, domestic violence, poor neighborhoods) and parenting [61].

### Limitations and Future Directions

This research has limitations that should be considered when interpreting the results. Although the study tested the instrument using an adequate sample in terms of size (*n* = 153) and socioeconomic variability, thus ensuring its statistical power, the numbers were not sufficient to provide normative data, and the age range of the children was limited to 18-month-old toddlers. A large study would be necessary for RIFL-P standardization and, therefore, for its clinical use.

The significance and novelty of this study was the development of a Brazilian Portuguese version of a psychometrically sound measure of responsive caregiving that requires little training (free, online), uses a short video clip of caregiver–child interaction (5 min), and takes little time to code (approximately 3 min per interaction). Thus, the adapted RIFL-P instrument represents a suitable measure for application in large-scale studies in Brazil to potentially impact policy, practice, and research.

## 5. Conclusions

The culturally adapted Brazilian Portuguese version of the RIFL-P measure proved to be a valid and reliable instrument for a brief assessment of responsive caregiving in parent–child interactions in Brazil. Efficient and valid measuring instruments of responsive caregiving appropriate to primary care settings are essential tools to advance ECD policies and programs. This study is one of the first of its kind, designed to provide a culturally adapted, reliable, and valid strengths-based measure of responsive parenting interactions with children under two years of age in Brazil.

## Figures and Tables

**Table 1:** Descriptive statistics of responsive interactions for learning items (English and Portuguese versions) in the Brazilian sample.

Item (English)	Item (Portuguese)	Brazilian Sample (*n* = 153)	
		*M*	*SD*
1. This parent gives clear and specific verbal directions.	1. Este cuidador dá instruções verbais claras e específicas.	3.27	1.5
2. This parent gives positive nonverbal directions.	2. Este cuidador dá instruções não verbais positivas.	2.87	0.85
3. This parent reminds the child about goals/rules of the task.	3. Este cuidador lembra a criança dos objetivos/regras da tarefa.	2.65	0.84
4. This parent will try to complete the task in a way that is sensitive to the child’s needs and desires.	4. Este cuidador procura completar a tarefa de maneira sensível às necessidades e aos desejos da criança.	2.71	0.88
5. This parent will try to follow the rules in a way that is sensitive to the child’s needs and desires.	5. Este cuidador procura seguir as regras de maneira sensível às necessidades e aos desejos da criança.	2.35	1.01
6. This parent is clear in his/her requests for help.	6. Este cuidador pede ajuda de maneira clara.	2.75	0.91
7. This parent is *sensitively* responsive to the child’s requests for help, even those that are subtle/nonverbal.	7. Este cuidador responde *sensivelmente* aos pedidos de ajuda da criança, mesmo os sutis e/ou não verbais.	2.44	0.9
8. This parent is good at rephrasing what the child does not understand.	8. Este cuidador consegue reformular instruções que a criança não entende.	1.98	1.06
9. This parent is sensitive to what the child knows and/or understands.	9. Este cuidador é sensível ao que a criança sabe e/ou compreende.	2.43	0.86
10. This parent gives positive feedback to reinforce the child.	10. Este cuidador oferece *feedback* positivo para reforçar o comportamento da criança.	2.96	1.37
11. This parent promotes turn taking between himself/herself and the child.	11. Este cuidador incentiva a alternância na interação com a criança.	2.41	0.88

*Note:* M = mean; SD = standard deviation.

**Table 2:** Item loadings for the Responsive Interactions for Learning measure in the Brazilian sample.

Scale/Item	Standardized Factor Loadings	R^2^
1. This parent gives clear and specific verbal directions.	0.61	0.37
2. This parent gives positive nonverbal directions.	0.76	0.57
3. This parent reminds the child about goals/rules of the task.	0.79	0.62
4. This parent will try to complete the task in a way that is sensitive to the child’s needs and desires.	0.89	0.80
5. This parent will try to follow the rules in a way that is sensitive to the child’s needs and desires.	0.90	0.81
6. This parent is clear in his/her requests for help.	0.89	0.79
7. This parent is *sensitively* responsive to the child’s requests for help, even those that are subtle/nonverbal.	0.87	0.75
8. This parent is good at rephrasing what the child does not understand.	0.83	0.68
9. This parent is sensitive to what the child knows and/or understands.	0.85	0.73
10. This parent gives positive feedback to reinforce the child.	0.65	0.42
11. This parent promotes turn taking between himself/herself and the child.	0.69	0.48

*Note:* All standardized factor loadings are significant at the 0.001 level. **R^2^** = coefficient of determination.

## Data Availability

The data that support the findings of this study are available from the corresponding author, upon reasonable request.

## Appendix A. RIFL-P Coding Sheet (Brazilian Portuguese Version)

**    Interações Responsivas para a Aprendizagem**

**  Codificação de Interações Breves em Duplas Cuidador-Criança**

- Responda às perguntas abaixo com base na interação deste cuidador especificamente com esta criança.
- Pontue rapidamente, de acordo com suas reações iniciais e impressões gerais; não se detenha demais em nenhum item.
- Use todas as informações disponíveis, incluindo as não verbais, para formar sua reação e impressões.
- Tente usar toda escala de 5 pontos, não deixe itens em branco (dê a cada item seu melhor palpite/avaliação).
- Observe somente por 5 min. Se ultrapassar 5 min, interrompa.

**Dê sua impressão de como este cuidador interage com a criança no dia-a-dia, com base no que você observou:**	**(Nada verdadeiro/Discordo totalmente)**		**(Algumas vezes verdadeiro/Concordo parcialmente)**	**(Muito verdadeiro/** **Concordo totalmente)**	
**Clareza na Comunicação**					
1. Este cuidador dá instruções verbais claras e específicas.	1	2	3	4	5
2. Este cuidador dá instruções não verbais positivas.	1	2	3	4	5
3. Este cuidador lembra a criança dos objetivos/regras da tarefa.	1	2	3	4	5
4. Este cuidador procura completar a tarefa de maneira sensível às necessidades e aos desejos da criança.	1	2	3	4	5
5. Este cuidador procura seguir as regras de maneira sensível às necessidades e aos desejos da criança.	1	2	3	4	5
6. Este cuidador pede ajuda de maneira clara.	1	2	3	4	5
**Leitura do Pensamento**					
7. Este cuidador responde *sensivelmente* aos pedidos de ajuda da criança, mesmo os sutis e/ou não verbais.	1	2	3	4	5
8. Este cuidador consegue reformular instruções que a criança não entende.	1	2	3	4	5
9. Este cuidador é sensível ao que a criança sabe e/ou compreende.	1	2	3	4	5
**Desenvolvimento da Mutualidade**					
10. Este cuidador oferece *feedback* positivo para reforçar o comportamento da criança.	1	2	3	4	5
11. Este cuidador incentiva a alternância na interação com a criança.	1	2	3	4	5

## Appendix B. RIFL-P Manual (Brazilian Portuguese Version)

**     Interações Responsivas para a Aprendizagem**

**   Codificação de Interações Breves em Duplas Cuidador-Criança**

- Responda às perguntas abaixo com base na interação do cuidador especificamente com esta criança.
- Pontue rapidamente, de acordo com suas reações iniciais e impressões gerais; não se detenha demais em nenhum item.
- Use todas as informações disponíveis, incluindo as não verbais, para formar sua reação e impressões.
- Tente usar toda a escala de 5 pontos, não deixe itens em branco (dê a cada item seu melhor palpite/avaliação).

**Escala Likert de 5 pontos**	**1 = Nada verdadeiro/Discordo totalmente**
	**2**
	**3 = Pouco verdadeiro/Concordo**
	**4**
	**5 = Muito verdadeiro/Concordo totalmente**

**CLAREZA NA COMUNICAÇÃO**
**1. Este cuidador dá instruções verbais claras e específicas.**
Este item se refere a comandos verbais com informações especificas e não genéricas. O cuidador dá informações suficientes para que a criança complete a tarefa, sem ser vago ou ambíguo.
**Exemplos:** “Coloque o bloco azul grande ao lado do bloco amarelo pequeno”; “Conte quatro círculos e depois coloque aqui”; “É o bloco verde claro”; “Vamos separar os blocos, você pega os azuis claros e eu pego os azuis escuros”; **ao invés de** “Coloque esse lá”; “Me dê aquele”; “Separe os blocos.”
**2. Este cuidador dá instruções não verbais positivas.**
Este item se refere ao uso de ações físicas para comunicar o que a criança deve fazer a seguir, incluindo também a modelagem. As ações são consideradas “positivas” na medida em que promovem a realização da tarefa de forma colaborativa, não agressiva e não hostil.
**Exemplos:** “Guiar a mão da criança, apontar para o lugar correto, apontar para a figura, apontar para um objeto e sugerir que a criança o pegue, servir de modelo, modelar (“Olha o que a mamãe vai fazer” ou “Olha só [e mostra como a criança deve fazer]”).
**3. Este cuidador lembra a criança dos objetivos/regras da tarefa.**
O cuidador cria um contexto para a atividade, comunicando à criança a ideia geral da tarefa, os objetivos e os passos a seguir.
**Exemplos:** “Lembra que nós estamos tentando copiar essa figura”; “A gente quer construir uma casa”; “Nós queremos colocar todos os blocos azuis para baixo”; “Agora vamos montar o braço”; “Não esqueça que só temos 5 min”.
**4. Este cuidador procura completar a tarefa de maneira sensível às necessidades e aos desejos da criança.**
Este item descreve a clareza com que o cuidador comunica à criança que há uma tarefa a ser feita na qual ele está engajado, estimulando assim a participação da criança. O cuidador demonstra envolvimento na atividade e esforço em completá-la. Se a criança parece não estar muito interessada na tarefa ou demonstra dificuldade, o cuidador procura contemplar com sensibilidade tanto os desejos/necessidades da criança quanto o objetivo da tarefa. Não é necessário que a atividade seja realizada corretamente, mas o cuidador deve tentar seguir a estrutura da tarefa. Um cuidador que desconsidera as necessidades da criança para completar a tarefa terá pontuação mais baixa nesse item, assim como um cuidador que não tenta modelar a estrutura e os objetivos da atividade. Um cuidador que leva em conta as necessidades da criança e também as exigências da tarefa deverá ter pontuação mais alta.
**Exemplos:** Seguir o ritmo da criança, fornecer uma orientação completa e abrangente, demonstrar paciência quando a criança está tentando realizar a tarefa, reformular as instruções quando a criança não entende.
**5. Este cuidador procura seguir as regras de maneira sensível às necessidades e aos desejos da criança.**
O cuidador segue as instruções do entrevistador, incluindo quaisquer regras mencionadas (p. ex., tocar nas cores certas), levando em consideração as necessidades da criança. Se a criança não compreende, então o cuidador explica as regras. Se a criança não demonstra interesse em seguir as regras, o cuidador procura de maneira sensível fazer com que a criança siga as regras. Um cuidador que desconsidera as necessidades da criança para seguir rigidamente as regras terá pontuação mais baixa nesse item. Um cuidador que leva em conta as necessidades da criança e também as regras da tarefa deverá ter pontuação mais alta.
**Exemplos:** O cuidador segue as regras da tarefa, lembra a criança sobre as regras, o cuidador é flexível quando a criança insiste em não seguir as regras e tenta fornecer oportunidades de aprendizagem.
**6. Este cuidador pede ajuda de maneira clara.**
O cuidador se comunica de uma forma que a criança consegue compreender. Este item não se refere especificamente a instruções verbais ou não verbais, mas à clareza geral da comunicação.
**LEITURA DO PENSAMENTO**
**7. Este cuidador responde *sensivelmente* aos pedidos de ajuda da criança, mesmo os sutis e/ou não verbais.**
Quando a criança demonstra precisar de ajuda (de modo verbal ou não verbal), o cuidador percebe e responde adequadamente. O cuidador não ignora sinais sutis ou óbvios de que a criança precisa de ajuda. Esse item *não* inclui respostas em tom irritado, frustrado ou hostil.
**Exemplos:** [A criança não consegue achar o bloco] “Esse está difícil de encontrar, não é?”; [A criança não consegue achar o bloco] “Olha o bloco ali!”; [puxa para perto o bloco que a criança está procurando].
**8. Este cuidador consegue reformular instruções que a criança não entende.**
Quando a criança tem dificuldade em entender as instruções ou a tarefa, o cuidador percebe e ajusta a linguagem para que ela compreenda melhor.
**Exemplos:** O cuidador percebe que a criança não está conseguindo achar o bloco amarelo pequeno, e diz “Procure o que tem só 4 círculos”; [ao construir o robô] a criança não responde quando o cuidador diz “Vamos começar de baixo” e o cuidador reformula dizendo “De que cor são os pês do robô?”
**9. Este cuidador é sensível ao que a criança sabe e/ou compreende.**
O cuidador é capaz de avaliar o nível de compreensão da criança e que tipo de ajuda ela precisa em cada tarefa, identificando a zona de desenvolvimento proximal (isto é, o nível em que as instruções são mais proveitosas para a criança - nem muito fáceis, nem muito difíceis).
**Exemplos:** Divide a tarefa em partes menores, usa linguagem adequada para o nível de desenvolvimento da criança, dá instruções básicas e apropriadas, percebe quando a criança não compreende algo, incentiva a independência da criança quando apropriado.
**DESENVOLVIMENTO DA MUTUALIDADE**
**10. Este cuidador oferece feedback positivo para reforçar o comportamento da criança.**
O cuidador responde às ações da criança com afirmações, vocalizações e/ou comportamentos positivos.
**Exemplos:** “Muito bem!”, “É assim mesmo!”, “Beleza!”, “Isso mesmo!”, “Isso!”, “Êee!”, “Parabéns!”, “Que bonito!”; “Que legal!”; [bate palmas; balança a cabeça seguindo as ações da criança].
**11. Este cuidador incentiva a alternância na interação com a criança.**
Este item é codificado quando o cuidador incentiva a reciprocidade e a alternância na interação, verbalmente ou não, e de modo explícito (p. ex., “Agora é a sua vez”) ou sutil (p. ex., “O que a gente vai fazer agora?”).
**Exemplos:** “O próximo é você quem faz”; “Agora é a sua/minha vez”; [apontar para a criança para indicar que é a vez dela]; “Onde será que esse bloco vai... [estimulando a criança a dar sua opinião]”. Outros exemplos incluem: “Agora a mãe coloca… agora a Maria coloca”; “A mãe vai botar primeiro e depois tu tens que fazer igual”; [A criança colocou a peça na parte de cima do pino e a mãe diz] “A mãe empurra pra ti”; “Agora bota tu”. Em interações altamente recíprocas, isto pode ser sutil (p. ex., o cuidador se inclina levemente para trás e/ou olha para a criança quando termina a sua vez).
